# Effects of the LASIK flap thickness on corneal biomechanical behavior: a finite element analysis

**DOI:** 10.1186/s12886-020-01338-8

**Published:** 2020-02-24

**Authors:** Lihua Fang, Yan Wang, Ruizhi Yang, Sijing Deng, Jiahao Deng, Linsun Wan

**Affiliations:** 1 Key Laboratory of National Engineering Laboratory for Nondestructive Testing and Optoelectric Sensing Technology and Application (Ministry of Education), Nanchang Hangkong University, Add: No 696. Fenghenan Rd, Donghu District, Nanchang city, Jiangxi Province 330063 China; 2 Tianjin Eye Hospital & Eye Institute, Ophthalmology and Visual Development Key Laboratory, Tianjin Medical University, Tianjin, 300020 China

**Keywords:** Biomechanical change, Finite element model, LASIK flap, Displacement, Stress

## Abstract

**Background:**

It is well known that the biomechanical properties change after LASIK refractive surgery. One reason is the impact of flap creation on the residual stroma. The results have revealed that the change is closely related with the flap thickness in several studies. However, the quantitative relationships between the distributions of displacement and stress on the corneal surface and flap thickness have not been studied. The aim of the study was to quantify evaluate the biomechanical change caused by the LASIK flap.

**Methods:**

By building a finite element model of the cornea, the displacement, the stress and the strain on the corneal surface were analyzed.

**Results:**

The results showed that the corneal flap could obviously cause the deformation of the anterior corneal surface. For example, the displacement of the corneal vertex achieved 15 μm more than that without corneal flap, when the thickness of corneal flap was 120 μm thick. This displacement was enough to cause the change of aberrations in the human eyes. In the central part of the cornea, the stress on the anterior corneal surface increased with flap thickness. But the change in the stress on the posterior corneal surface was significantly less than that on the anterior surface. In addition, the stress in the central part of the anterior corneal surface increased significantly as the intra-ocular pressure (IOP) increase. Furthermore the increase of IOP had a clearly less effect on stress distribution at the edge of the cornea. Distributions of strain on the corneal surface were similar to those of stress.

**Conclusions:**

The changes in the biomechanical properties of cornea after refractive surgery should not be ignored.

## Background

Modern excimer laser refractive surgery attempts to correct refractive errors by altering the shape of the front surface of the cornea [1]. Despite the great potential of refractive surgery, there are still many complications associated with it, such as post-LASIK corneal ectasia [2]. In fact, the surgical outcomes are not always as expected. Several risk factors have been identified for these outcomes, such as a large preoperative myopic refractive error, a thin residual stromal bed, or other corneal topographic abnormalities [3]. In fact, the impact of flap creation on the residual stroma most likely plays a critical role in induced ocular higher-order aberrations [4]. Biomechanical effects of the residual stroma after refractive surgery might also play a role in changes in the corneal curvature that do not represent a development of ectasia but that actually affect refractive outcome [5].

A finite element simulation of corneal biomechanical behavior can predict ophthalmic surgery and the material parameters of the cornea [6, 7]. Recent developments in wavefront analysis [8, 9], corneal material characterization, and topographic mapping offer extremely valuable information for numerical modeling. The finite element simulation is one of the cornerstones of the biomechanical approach to improving visual outcomes after surgery [10]; therefore, the finite element model (FEM) has been used to simulate the changes in mechanical properties caused by the LASIK flap.

Theoretical simulation plays an important role in improving clinical practice. For example, a numerical model can be adopted in clinical practice to plan and optimize refractive surgeries [11, 12]. FEM can be a useful tool for providing insight into the effects of such alterations of material properties of the geometry and optical performance of the human eye. Here, we are attempted to assess the biomechanical changes caused by the LASIK flap in FEM.

The purposes of this study were to (1) analyze the corneal biomechanical properties as a response to changes in flap thickness; (2) investigate the behavior of the cornea under different loading states and improve the predictions of the mechanical response to refractive surgery, and (3) assess the distribution of stress and strain on the corneal surface, especially the nonuniform distribution of stress.

## Methods

### Geometric model of the cornea

The cornea consists of the following five distinct cell layers: outer epithelium; Bowman’s membranes; central stroma; Descemet’s membranes, and inner endothelium, where the stroma is an important part of the cornea. At the micro level, the stroma forms ~ 90% of the corneal thickness and is composed of thin collagen fibrils, corneal cells, and extracellular viscous matter. In general, the anterior and posterior surfaces of the normal human cornea are ellipsoidal. In this study, human cornea data, such as mathematical models of the anterior and posterior surfaces, are as follows:

Anterior corneal surface

$$ \frac{x^2}{R_x}+\frac{y^2}{R_y}+\frac{{\left(z-{R}_z\right)}^2}{R_z}=1, $$

In the equation, *R*_*x*_, *R*_*y*_ and *R*_*z*_ are the half-shaft of horizontal, vertical and longitudinal direction respectively on anterior corneal surface, which is about 7 to 8 mm.

Posterior corneal surface

$$ \frac{x^2}{R_x}+\frac{y^2}{R_y}+\frac{{\left(z-{R}_z-d\right)}^2}{R_z}=1, $$

In the equation, *R*_*x*_, *R*_*y*_ and *R*_*z*_ are the half-shaft of horizontal, vertical and longitudinal direction respectively on posterior corneal surface, which is about 6.7 to 7.6 mm. The *d* represents the central thinnest corneal thickness (CCT).

### Material properties

The cornea is somewhat incompressible tissue that shows nonlinear stress–strain characteristics [13]. In addition, the cornea is modeled as isotropic material, and the formula for hyperelastic material has been used to simulate its elastic properties; therefore, the behavior of the cornea material can be presumed to follow the Ogden Hyperelastic Material model. The strain energy potential can be expressed using the following equation [14]:

1 $$ \mathrm{W}=\sum \limits_{i=1}^N\frac{\mu_i}{\alpha_i}\left({\overline{\lambda}}_1^{\alpha_i}+{\overline{\lambda}}_2^{\alpha_i}+{\overline{\lambda}}_3^{\alpha_i}-3\right)+\sum \limits_{\mathrm{k}=1}^N\frac{1}{d_k}{\left(J-1\right)}^{2k} $$

Where W conveys the strain energy potential, ${\overline{\lambda}}_p$represents deviatoric principal stretches defined as ${\overline{\lambda}}_p={J}^{-\frac{1}{3}}{\lambda}_p$and *λ*_*p*_depicts the principal stretches of the left Cauchy–Green tensor, and *J* represents the determinant of the elastic deformation gradient.

$$ N,{\mu}_p,{\alpha}_p\; and\;{d}_p= material\ constants. $$

The initial shear modulus is defined as

$$ \mu =\frac{1}{2}\sum \limits_{i=1}^N{\alpha}_i{\mu}_{i,} $$

And the initial bulk modulus is defined as

$$ K=\frac{2}{d_1} $$

For the purposes of the current study, the experimentally derived stress-versus-strain data were fit to the above material model. The parameters μ_1_ and α_1_ are hyperelastic constants obtained from fitting the experimental stress-versus-strain data, with the values of 0.60415Mpa and 16.54, respectively [15]. The Poisson ratio values of the human cornea ranges from 0.42 to 0.5, which denotes that the cornea tissue exhibits a nearly incompressible behavior [16].

### The finite element model of the cornea

**Fig. 1:**
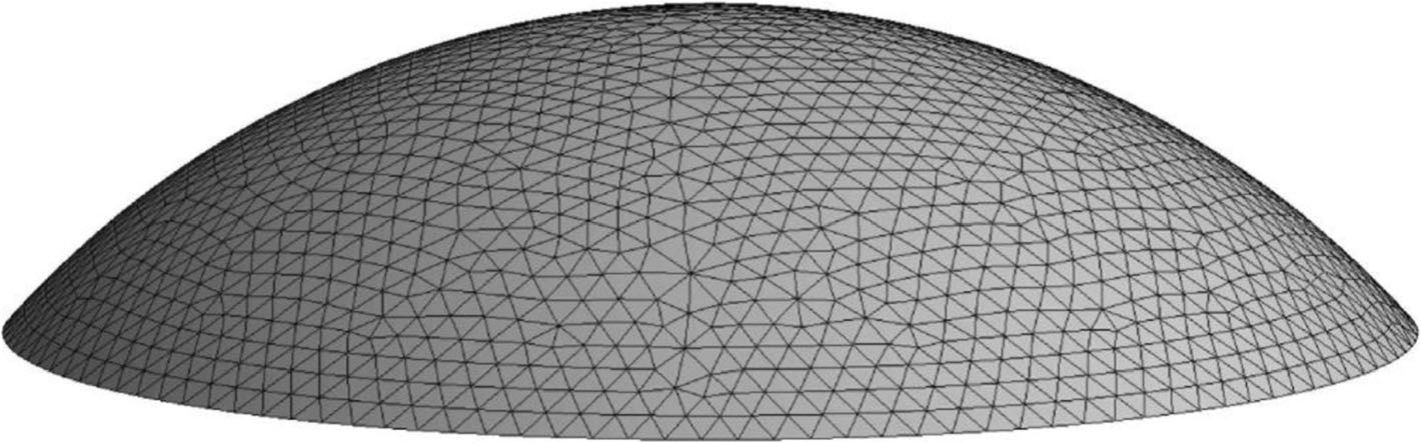
Finite element mesh of the original cornea

In light of the shape and characteristics of the cornea, the generation of hex dominant computational fluid dynamics (CFD) mesh was selected. The central thickness *d* of the cornea was ~ 0.55 mm [17]. In order to ensure both computational efficiency and simulation accuracy, when mesh density failed to achieve a significant change in nodal displacement we considered the model sufficiently resolved, the size of meshes was selected to be 0.20–0.30 mm. The “free orientation unit” was selected to more rationally and conveniently generate a satisfactory mesh. The corneal model comprised 5708 units and 9753 nodes after mesh generation, as shown in Fig. 1. For different corneal flap thicknesses, there were slight differences in the number of units and nodes included in the corneal model.

The corneal boundary and other tissues, such as the sclera, were restrained and firmly fixed by the surrounding biological tissues, including the ciliary processes and iris, as a shell of which the bottom edges are clamped; therefore, the restrained bottom interface of the cornea could be considered to be a clamped boundary.

### Simulation of corneal flaps

**Fig. 2:**
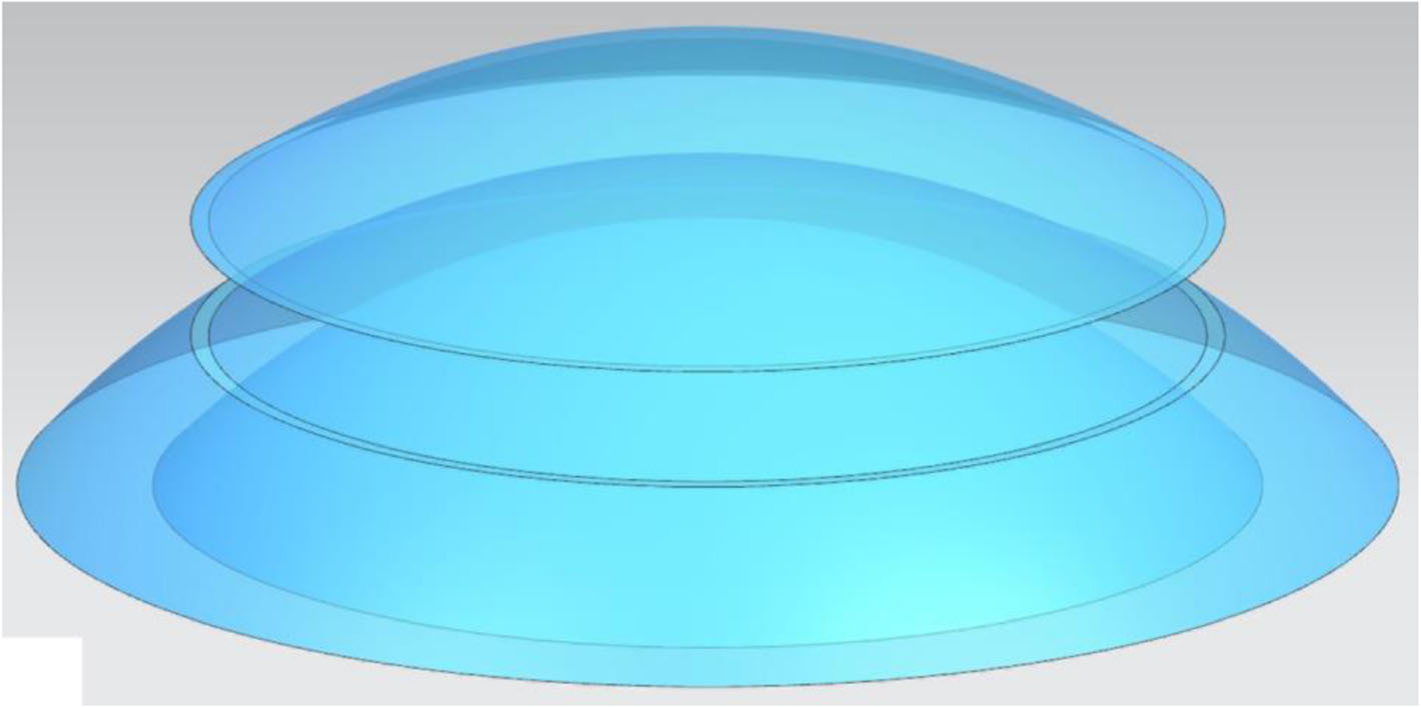
Corneal flap 8.1 mm in diameter and 120 μm thick

The corneal flap refers to a thin flap of corneal tissue that is cut during refractive surgery. In our study, the diameter of the corneal flap was 8.1 mm, and the thickness of the corneal flap was presumed to be 90, 120, 150, 180, 210, and 240 μm, respectively (Fig. 2).

### Corneal shape change and stress distribution

The changes in corneal shape refer mainly to the displacement of the corneal surface. The refractive power of a normal eye is mainly from the anterior corneal surface; therefore, only the displacement of the anterior corneal surface was studied here from two perspectives. First, we explored the relationship between displacement of the anterior corneal surface and the thickness of the corneal flap. The displacement of the anterior corneal surface referred to the difference between the displacement of the anterior corneal surface with and without the corneal flap under the same IOP. Second, we studied the relationship between displacement of the anterior corneal surface and IOP. The displacement of the anterior corneal surface was the difference between the displacements of the anterior corneal surface under different IOPs with the same corneal flap thickness. The stress distribution on the corneal surface was also studied from two aspects. First, the influence of changes in IOP on stress distribution on the corneal surface was assessed (i.e., the stress distribution on the anterior and posterior corneal surfaces under the same corneal flap thickness). Second, the influence of the thickness of the corneal flap on the stress distribution on the corneal surface was studied (i.e., stress distribution on the anterior and posterior corneal surfaces under the same IOP).

## Results

### Influence of the thickness of corneal flap on the displacement of the anterior corneal surface

**Fig. 3:**
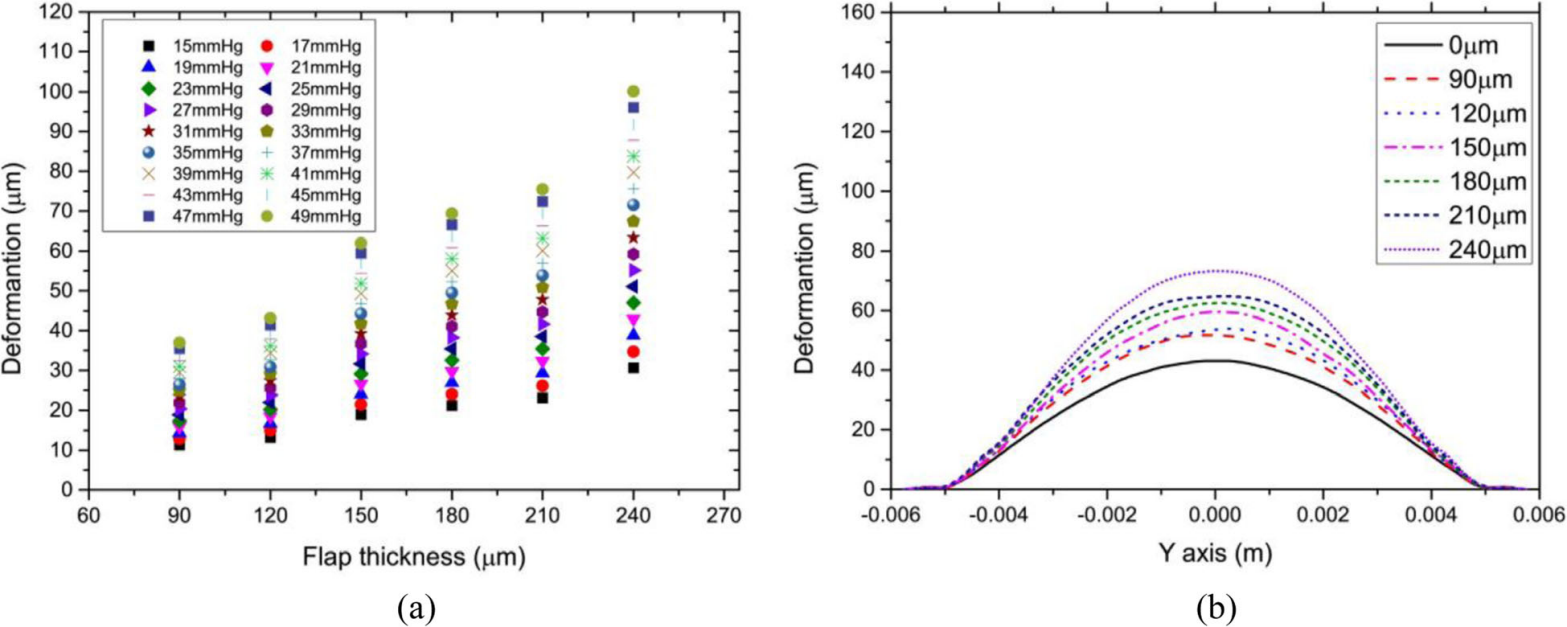
**a** Relationship between corneal vertex displacement and corneal flap thickness. Intraocular pressure ranged from 15 to 49 mmHg. **b** Relationship between the anterior corneal surface displacement along the Y-axis and corneal flap thickness. The corneal flap thickness was assumed to be 90, 120, 150, 180, 210, and 240 μm, respectively. The unit of corneal vertex displacement was μm

Figure 3 showed the relationship between the corneal vertex displacement and corneal flap thickness. To present more comprehensive corneal mechanical properties, the range of IOP was from 15 mmHg to 49 mmHg. In addition, we observed that displacement of the anterior corneal surface showed rotational symmetry; therefore, we studied the relationship between displacement of the anterior corneal surface along the Y-axis and the thickness of the corneal flap. In the study, IOP was constant at 17 mmHg, and the thickness of the corneal flap was set to 90, 120, 150, 180, and 210 μm, respectively.

As shown in Fig. 3(a), under the same IOP, there was an approximate linear relationship between corneal vertex displacement and IOP. For example, the change in corneal vertex displacement when the corneal flap was 150 μm thick was more significant than when the corneal flap was 120 μm thick. Similarly, the change in corneal vertex displacement when the flap was 240 μm thick was more significant than when the flap was 210 μm thick. As shown in Fig. 3(b), there was maximum displacement on corneal vertex, but the displacement of the flap edge was very small. The displacement of the anterior corneal surface showed a similar rotational symmetry. It was obvious that production of corneal flap could result in deformation of the anterior corneal surface. When the flap thickness was 240 μm, corneal vertex displacement was 30 μm larger than that when there was no corneal flap, which is enough to cause corneal aberrations.

### Effect of IOP on the displacement of the anterior corneal surface

**Fig. 4:**
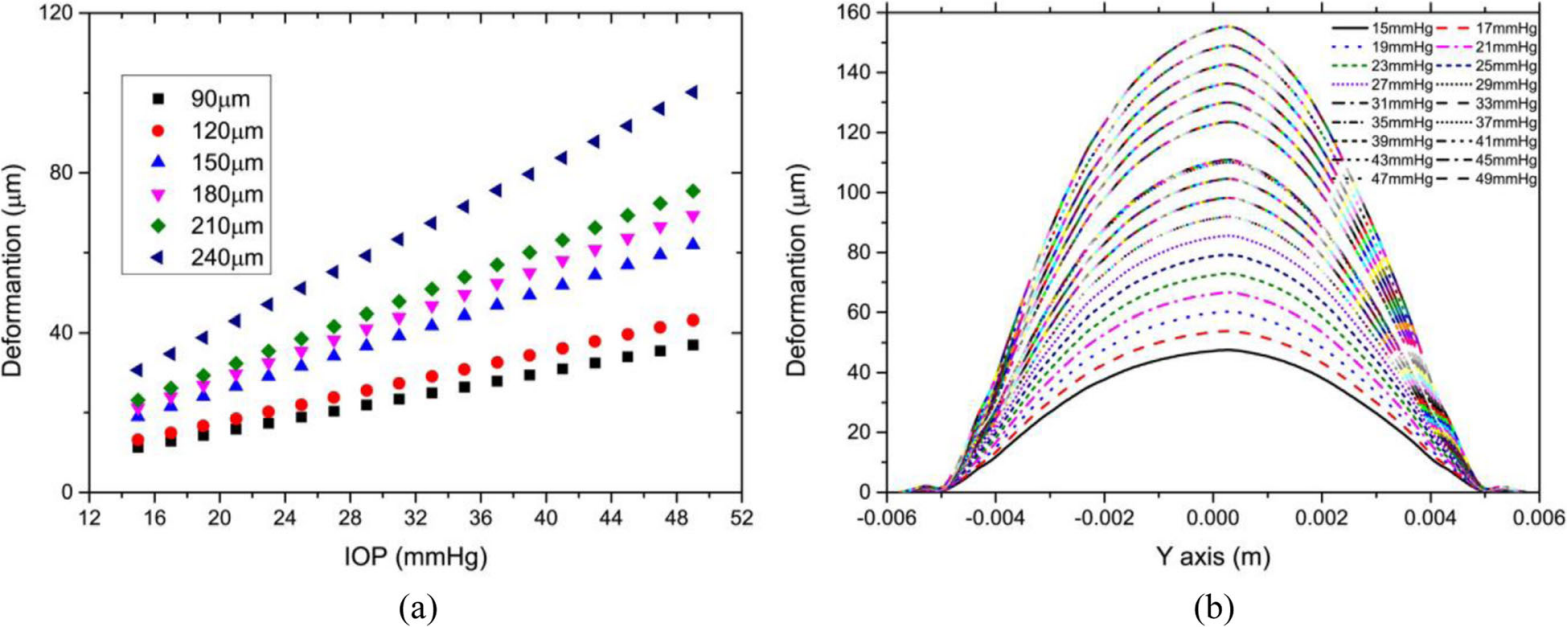
**a** The relationship between corneal vertex displacement and intraocular pressure. The flap thickness was set at 90, 120, 150, 180, 210, and 240 μm, respectively. The unit of the corneal vertex displacement was μm. **b** The relationship between displacement of the anterior corneal surface along the Y axis and intraocular pressure. IOP ranged from 15 to 49 mmHg

Figure 4 showed the relationship between displacement of the corneal vertex and IOP. To show more comprehensive mechanical properties of the cornea, the maximum IOP was up to 49 mmHg, and the maximum flap thickness was set to 240 μm. The relationship between the displacement of the anterior corneal surface along the Y-axis and IOP was studied. IOP was assumed to be 15–49 mmHg and flap thickness was assumed constant at 120 μm.

As shown in Fig. 4(a), there was a significant linear relationship between corneal vertex displacement and IOP. In other words, when IOP was less than 49 mmHg, the biomechanical behavior of the cornea showed a linear relationship. As shown in Fig. 4(b), when the flap thickness was constant, the displacement of the anterior corneal surface increased linearly with IOP.

### Influence of the thickness of corneal flap on the stress on the corneal surface

**Fig. 5:**
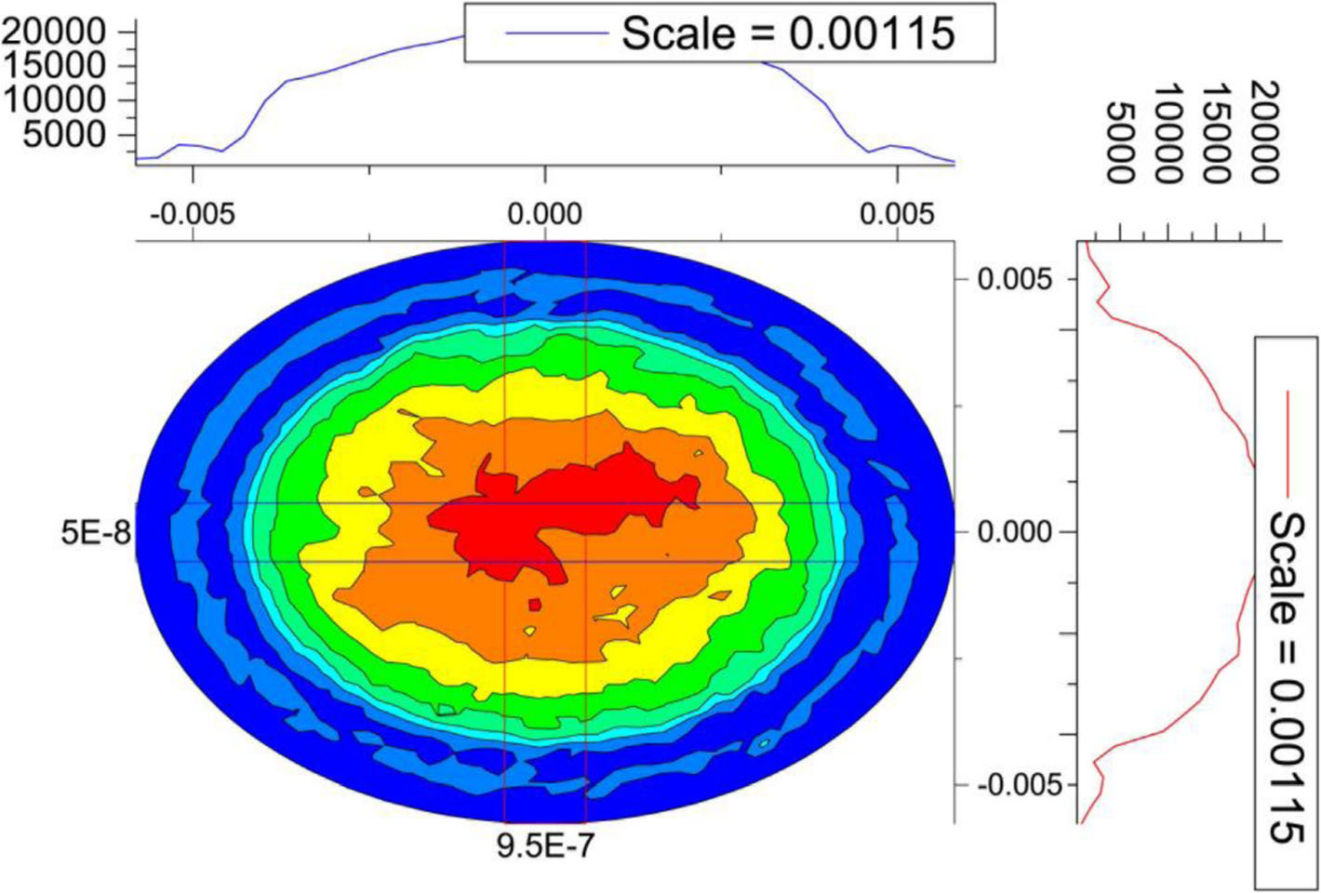
Contour map of the distribution of stress on the anterior corneal surface. Flap thickness was constant at 120 μm and the intraocular pressure was 17 mmHg

Figure 5 showed the two-dimensional distribution of stress on the anterior corneal surface.

As shown in Fig. 5, stress distribution on the corneal surface had a nearly, but not completely, rotational symmetry. In fact, the cover of eyelid also affected stress distribution. On the other hand, it could be seen from the skeleton map along the X- and Y-axes that stress distribution was basically consistent; therefore, in subsequent analyses, only the distribution of stress and strain along the Y-axis was analyzed.

**Fig. 6:**
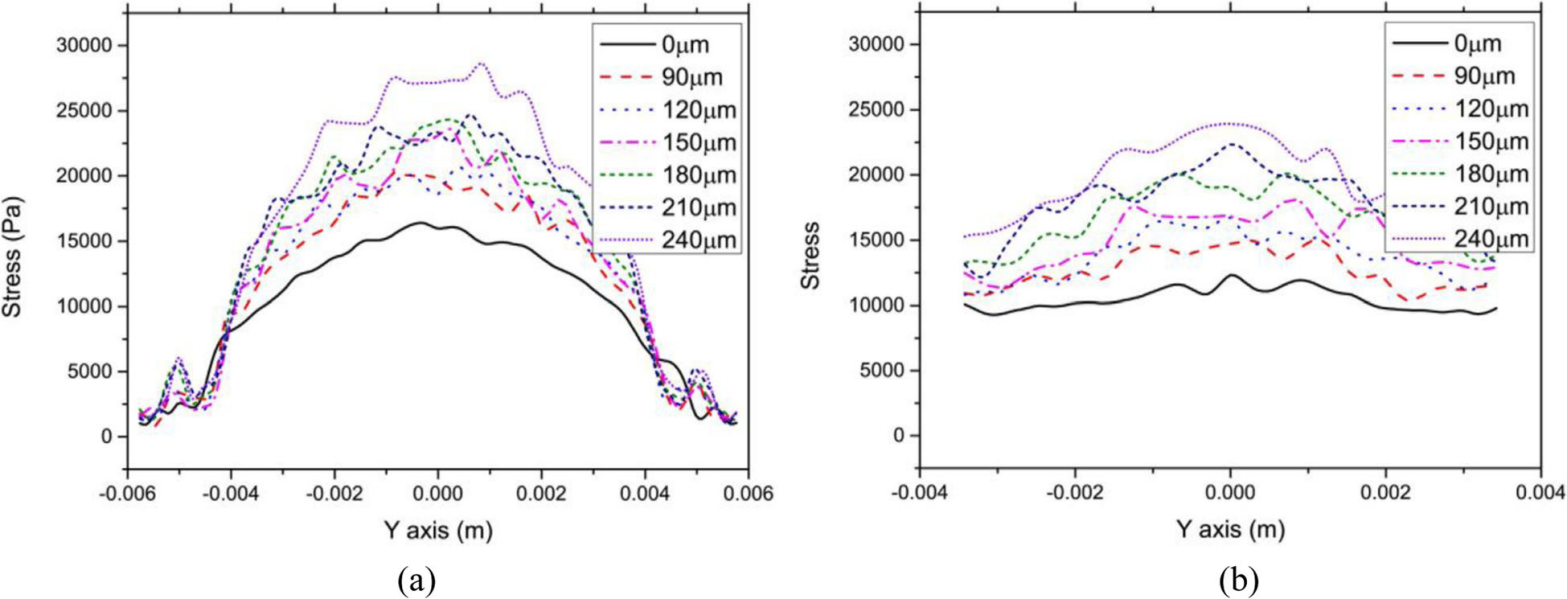
Relationship between stress on the anterior and posterior corneal surfaces along the Y-axis and flap thickness. The flap thickness was set to 90, 120, 150, 180, 210, and 240 μm, respectively. Intraocular pressure was constant at 17 mmHg. **a** Anterior surface. **b** Posterior surface

Figure 6 showed the relationship between stress on the anterior and posterior corneal surfaces along the Y-axis. IOP was constant at 17 mmHg. The flap thickness was set to 90, 120, 150, 180, and 210 μm, respectively.

As seen in Fig. 6(a), stress distribution on the corneal surface was not completely bilaterally symmetrical, but the stress on the corneal surface tended to be at the maximum at the corneal vertex; however, because of the fluctuation in stress distribution, the stress was not at a maximum at the corneal vertex, which might be the result of asymmetrical mesh generation. In fact, the thinnest point of the cornea was not at its center. Meanwhile, the asymmetry of the cornea could result in an asymmetrical distribution of stress. In the center of the cornea, the stress on the anterior corneal surface increased with corneal flap thickness, but the stress on the corneal edge did not significantly change. Moreover, the stress fluctuates slightly if there was no corneal flap. Nevertheless, stress fluctuated more significantly with an increase in corneal flap thickness. Finally, stress increased significantly around the incision in the corneal flap. The results indicated that both the incision and thickness of the corneal flap had an obvious effect on stress distribution on the cornea.

As shown in Fig. 6(b), changes in stress on the posterior surface with the Y value were not more significant than those on the anterior surface. The stress on the interior corneal surface increased with corneal flap thickness, and stress fluctuated more significantly. At the same time, the incision in the corneal flap had nearly no influence on the stress on the posterior corneal surface.

### Effect of the IOP on the stress on the corneal surface

**Fig. 7:**
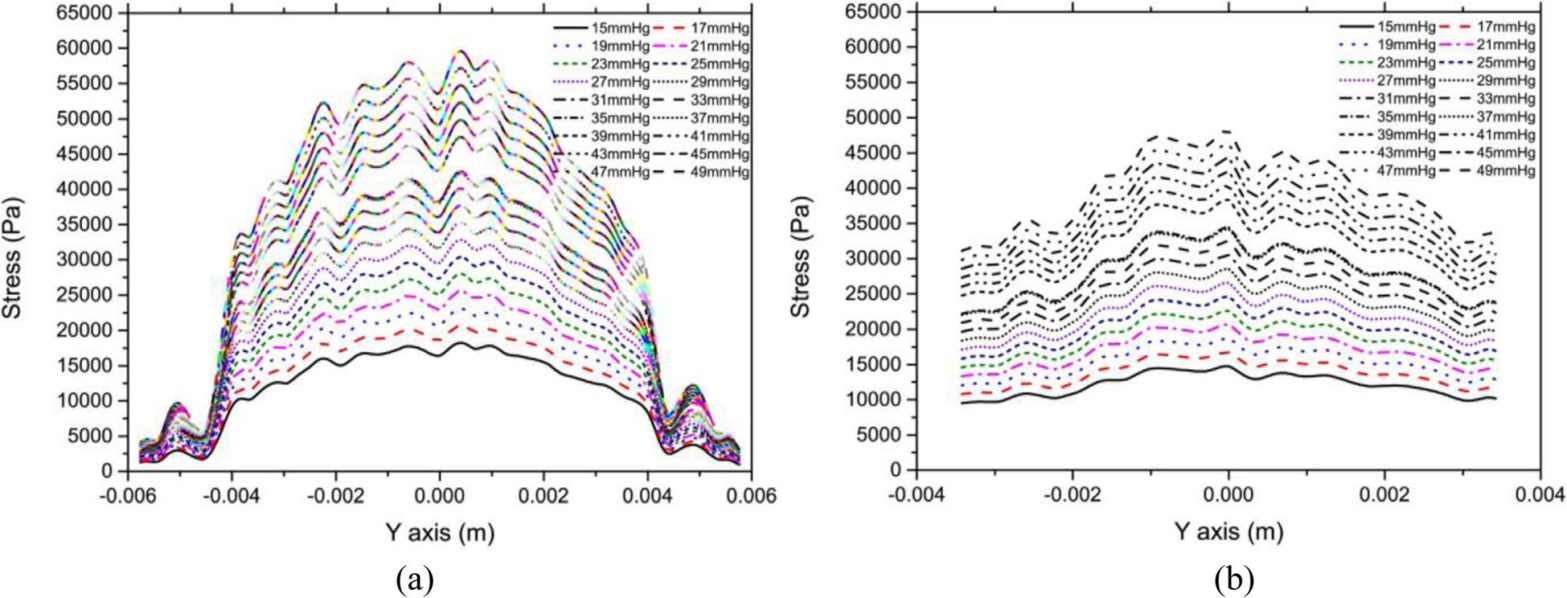
Relationship between stress on the anterior and posterior corneal surfaces along the Y-axis and IOP. IOP was set to 15–49 mmHg. The corneal flap thickness was constant at 120 μm. **a** Anterior surface. **b** Posterior surface

Figure 7 showed the relationship between stress on the anterior and posterior corneal surfaces along the Y-axis and IOP. IOP was set to 15–49 mmHg, and the corneal flap thickness was constant at 120 μm.

As shown in Fig. 7(a), the stress on the center of the anterior corneal surface increased significantly with IOP, but the increase in IOP at the corneal edge had a less-significant influence on the stress. In addition, there was nearly no change in stress near the corneal flap with the increase in IOP.

From Fig. 7(b), we observed that stress on the center of the posterior surface increased with IOP, but the increase was much less significant than that of the anterior surface. The stress on the edge of the posterior surface increased with IOP, but the increase was much less significant than that of the center. In addition, there was no sharp change in stress distribution on the incision of the corneal flap.

### Influence of the thickness of corneal flap and IOP on strain on the corneal surface

**Fig. 8:**
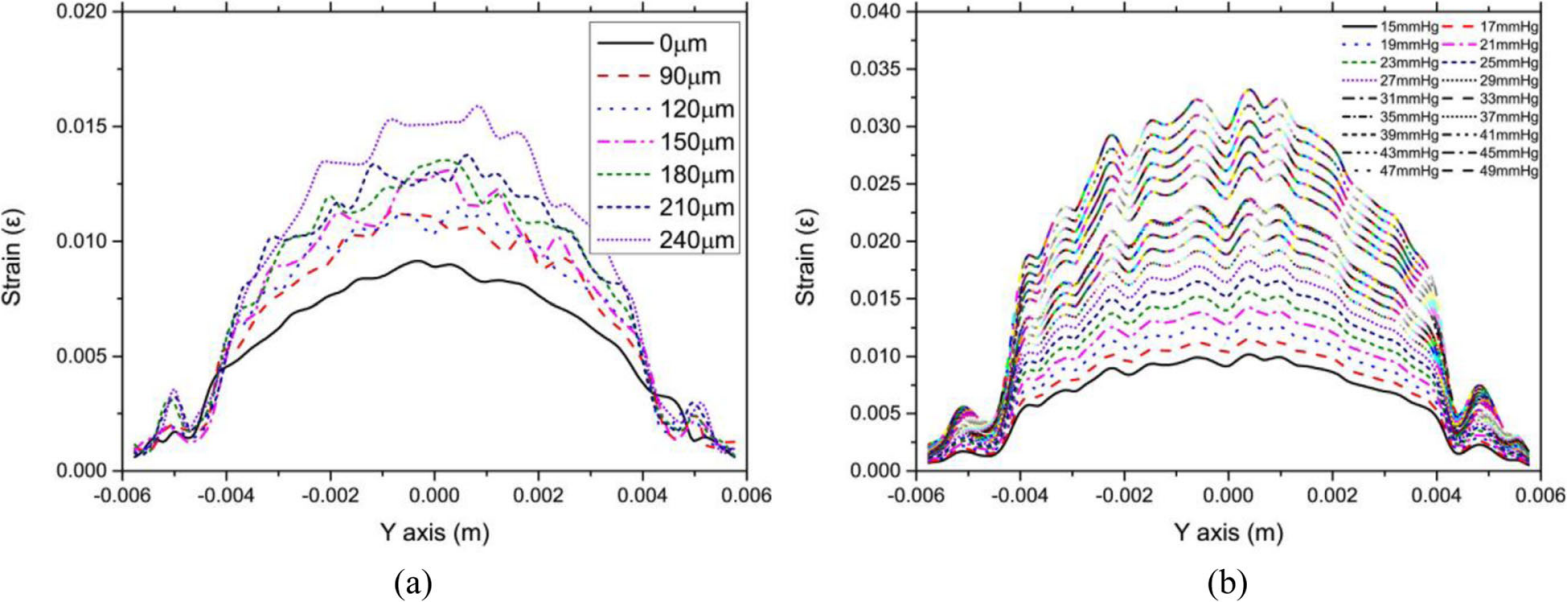
**a** Relationship between the strain on the anterior corneal surface along the Y-axis and corneal flap thickness. The corneal flap thickness was set to 90,120, 150, 180, 210, and 240 μm, respectively. The intraocular pressure (IOP) was constant at 17 mmHg. **b** Relationship between strain on the anterior corneal surface along the Y-axis and corneal flap thickness. IOP was set to 15–49 mmHg. Corneal flap thickness was constant at 120 μm

Figure 8(a) showed the relationship between strain on the anterior corneal surface along the Y-axis and corneal flap thickness. IOP was constant at 17 mmHg. Corneal flap thickness was set to 90, 120, 150, 180, 210, and 240 μm, respectively. Figure 8(b) showed the relationship between strain on the anterior corneal surface along the Y-axis and IOP. IOP was set to 15–49 mmHg, and corneal flap thickness was constant at 120 μm.

As shown in Fig. 8(a), the strain distribution on the corneal surface was similar to that of the stress distribution. In the center of the cornea, the strain on the anterior corneal surface increased with corneal flap thickness, but there was no significant change in the strain on the corneal edge. It could be seen from Fig. 8(b) that the strain on the center of the anterior corneal surface increased significantly with IOP, but the strain on the corneal edge was less-significantly influenced by IOP. Moreover, there was nearly no change in the strain near the corneal flap with an increase in IOP.

## Discussion

The creation of corneal flaps had a significant impact on the biomechanical properties of the cornea. Corneal hysteresis changes had been investigated using an ocular response analyzer, and the results had shown that thicker flaps presented a greater biomechanical impact on the cornea [18]. Deenadayalu et al. [19] demonstrated that the depth of the lenticular cut was a significant parameter that was tested at varying depths from 0.24 D at 100 μm to 1.25 D at 275 μm; however, in the study, the displacement of the corneal surface and the distribution of stress and strain were explored. The results also indicated a linear relationship between the displacement of the anterior corneal surface and corneal flap thickness. These differences in results could be attributed to a different Young modulus. Medeiros et al. [18] revealed that biomechanical effects might also be important in explaining the changes in the curvature of the residual stroma after flap creation and photoablation that did not represent ectasia but that, nonetheless, had an effect on refractive outcome. Uzbek et al. [20] demonstrated that flap creation with the IntraLase laser produced a biomechanical consequence consistent with reduction in corneal stiffness. One limitation to our study was that the actual corneal cutting depth during corneal refractive surgery was not considered. In fact, the actual ablation profile was related to the refractive surgery procedure. The refractive surgery procedures included conventional refractive surgery, wave-front aberration-guided surgery, SMILE (small-incision lenticule extraction) surgery and so on. In this study, only the effect of the corneal flap on biomechanics was considered. Another limitation of the study was that flap hinge was not taken into account. Actually the position and size of the flap hinge had influence on the result of refractive surgery. In addition, the result of obvious non-rotational symmetry would be shown due to the existence of flap hinges. The effect of different refractive surgery procedures and the position and size of the flap hinge on corneal biomechanics would be studied in the future.

In addition, displacement of the anterior corneal surface included displacement along the X-, Y-, and Z-axes. The analysis revealed that the displacement along the Z-axis was dominant (~ > 90%); therefore, displacement of the anterior corneal surface along the Z-axis was considered.

Our study indicated that stress distribution did not cause an obvious rotational symmetry, but fluctuated significantly, even near the corneal vertex. This could be because stress on the surface units must be different from that on the interior units, while stress on the anterior and posterior corneal surfaces was discussed. Meanwhile, mesh generation and mesh asymmetry also affected stress distribution on each mesh unit. In addition, stress on the corneal flap incision did not significantly increased, while an increase in stress on the corneal edge might be caused by the fixed and restrained corneal under surface. Finally, the creation of corneal flaps could significantly increase stress on the cornea. For example, when IOP was 17 mmHg and the flap was 120 μm thick, the maximum stress was 20,678 Pa, while it was 16,398 Pa before the creation of the corneal flap, an increase of 26.1%. In addition, when IOP was 49 mmHg and the flap was 120 μm thick, the maximum stress was 59,603 Pa, while it was 47,265 Pa before the creation of the corneal flap, an increase of 26.1% also. The significant increase in stress remarkably influenced the subsequent corneal shape.

Although the biomechanical mechanisms for creating the flap in LASIK refractive surgery had been studied, FEM relied strongly on the mechanical properties that were assigned to the corneal tissue and some assumptions within the model. A better understanding of the biomechanical response of the cornea should be provided by a sophisticated corneal biomechanical model, including different cutting depths and optical zone sizes; therefore, a more accurate prediction of the post-surgery results would be available.

Our results showed that IOP had distinctly influence on the corneal vertex displacement, the distribution of stress and the distribution of strain. In particular, the corneal vertex displacement and the distribution of stress on the center of the corneal surface increased significantly with IOP, but those at the corneal edge changed less-significant significantly. This might be an important source of the instability in the results of refractive surgery.

In this study, the biomechanical effects after creating the flap resulted in a hyperopic shift. In fact, for the clinical refractive surgery system, the main goal is to correct the refractive error. Therefore, the residual refractive error was small in most clinical measurements [21, 22]. Hondur et al. found that more than 90% of eyes were within ±0.50 D of emmetropia in 12 months [22]. But individual residual refractive error may be ±1D or more. This may be partly due to the effects of biomechanics.

Furthermore, the distribution of strength throughout the cornea may play an important role. However, as far as I know, there were no the clinical measurements for the distribution of strength. Additionally, the distribution was particularly useful for the analyses of individual human eye. Once the distribution was measured in vivo in clinical, the influence of the distribution on biomechanics could be analyzed.

## Conclusion

The corneal flap could obviously cause a deformation of the anterior corneal surface. For example, the displacement of the corneal vertex was 15 μm more than that without the corneal flap when the corneal flap was 120 μm thick. The issue of cutting depth in refractive surgery was not considered in our study and would be explored in the future. This displacement was enough to cause aberrations in the human eye. With a finite flap thickness, the displacement of the anterior corneal surface increased linearly with IOP. In the central part of the cornea, the stress on the anterior corneal surface increased with flap thickness, but there was no significant change in the stress on the corneal edge. The change in the stress on the posterior corneal surface was significantly less than that on the anterior surface. There was a significant increase in the stress around the incision in the corneal flap. In addition, the stress in the central part of the anterior corneal surface increased significantly as IOP increased, but the IOP increase had a clearly less effect on stress distribution at the edge of the cornea. Distributions of strain on the corneal surface were similar to those of stress; therefore, the changes in the biomechanical properties of the cornea after refractive surgery could not be ignored.

## Data Availability

All data generated or analyzed during this study are included in this published article.

## Abbreviations

CCT: central thinnest corneal thickness
CFD: computational fluid dynamics
FEM: finite element model
IOP: intra-ocular pressure
